# Alterations in Ovarian Cancer Cell Adhesion Drive Taxol Resistance by Increasing Microtubule Dynamics in a FAK-dependent Manner

**DOI:** 10.1038/srep09529

**Published:** 2015-04-17

**Authors:** Daniel J. McGrail, Niti N. Khambhati, Mark X. Qi, Krishan S. Patel, Nithin Ravikumar, Chandler P. Brandenburg, Michelle R. Dawson

**Affiliations:** 1 School of Chemical & Biomolecular Engineering, Georgia Institute of Technology, Atlanta, GA; 2 School of Chemistry & Biochemistry, Georgia Institute of Technology, Atlanta, GA; 3 The Petit Institute for Bioengineering and Bioscience, Georgia Institute of Technology, Atlanta, GA

## Abstract

Chemorefractory ovarian cancer patients show extremely poor prognosis. Microtubule-stabilizing Taxol (paclitaxel) is a first-line treatment against ovarian cancer. Despite the close interplay between microtubules and cell adhesion, it remains unknown if chemoresistance alters the way cells adhere to their extracellular environment, a process critical for cancer metastasis. To investigate this, we isolated Taxol-resistant populations of OVCAR3 and SKOV3 ovarian cancer cell lines. Though Taxol-resistant cells neither effluxed more drug nor gained resistance to other chemotherapeutics, they did display increased microtubule dynamics. These changes in microtubule dynamics coincided with faster attachment rates and decreased adhesion strength, which correlated with increased surface β1-integrin expression and decreased focal adhesion formation, respectively. Adhesion strength correlated best with Taxol-sensitivity, and was found to be independent of microtubule polymerization but dependent on focal adhesion kinase (FAK), which was up-regulated in Taxol-resistant cells. FAK inhibition also decreased microtubule dynamics to equal levels in both populations, indicating alterations in adhesive signaling are up-stream of microtubule dynamics. Taken together, this work demonstrates that Taxol-resistance dramatically alters how ovarian cancer cells adhere to their extracellular environment causing down-stream increases in microtubule dynamics, providing a therapeutic target that may improve prognosis by not only recovering drug sensitivity, but also decreasing metastasis.

Ovarian cancer is a leading cause of cancer-related deaths in women. Due to lack of early detection techniques, more than 75% of the patients are diagnosed after the cancer spreads from the primary site^1^. Though tumor debulking has proven extremely beneficial for patient survival^2^, the successful use of chemotherapeutics is still critical to target the disseminated disease. The standard treatment protocol of tumor resection followed by dual agent chemotherapy consisting of platinum therapy plus Taxol (paclitaxel) have increased progression-free survival to nearly 18 months and overall survival to 38 months, though once the cancer returns it is often no longer sensitive to these chemotherapeutic agents^3^. At this point, the disease rapidly progresses with progression-free survival of 3–5 months and overall survival rarely exceeding a year even with new experimental treatments^4^. Thus, there exists a clear clinical need for improved understanding of recurrent disease.

This chemotherapeutic resistance could occur through a variety of mechanisms, such as increased drug efflux, increased survival signals, blocking of death signals, or even changes in cell tubulin by either binding site mutations and expression of different isoforms^5^,^6^. To understand these mechanisms a series of pioneering studies have been performed by isolating Taxol-resistant populations of cancer cell lines and comparing them to their parental controls verifying many of these changes take place including over expression of the P-glycoprotein (Pgp) drug efflux pump^7^, alterations in stress response and survival cascades^8^, increased microtubule dynamics^9^, alteration in expression of tubulin isoforms^10^, and mutations to Taxol binding sites^11^. Of these studies, the former two were carried out on cells isolated to have super-physiological resistance to Taxol with IC_50_ values in excess of 1 μM. In contrast, the latter studies that observed altered microtubules isolated cells by slowly ramping the Taxol concentration to nanomolar concentrations relevant in the clinic^12^ produced an IC_50_ of approximately 20–45 nM, over two orders of magnitude more sensitive.

Though chemoresistance is a key hurdle in the treatment of ovarian cancer, the majority of cancer deaths are ultimately caused by metastatic spread of the disease to distant sites^13^. Ovarian cancer typically shows extensive metastasis following treatment with Taxol, and Taxol-resistant cell lines are more metastatic in mouse xenograph models^14^. In order to metastasize, ovarian cancer cells must first detach from the primary tumor. After detaching, the cells disseminate through the peritoneal cavity before re-adhering to a secondary site, often the omentum. This adhesion represents the first rate-limiting step in ovarian cancer progression^15^. Cell adhesion is controlled both by extracellular integrin domains which bind to the extracellular matrix (ECM), as well as intracellular focal adhesion adapter proteins such as paxillin and vinculin which act as linkers between the transmembrane integrins and internal cytoskeleton^16^. Integrin expression has been linked to progression in a variety of cancers, causing increased metastasis, increased tumor survival, and decreased patient prognosis^17^. In ovarian cancer, expression of β1 integrin has been linked to ovarian cancer invasion and metastasis^18^,^19^. Additionally, specific integrin heterodimers α4β1 and αvβ5 have been shown to increase metastasis and proliferation, respectively^20^,^21^. Moreover, previous studies have demonstrated a bi-directional link between adhesion signaling and the microtubule dynamics targeted by Taxol where focal adhesion signaling can alter microtubules^22^,^23^ and microtubules can alter adhesion dynamics^24^. Based on this, we hypothesized that ovarian cancer resistance to Taxol may lead to alterations in adhesion dynamics, which may contribute to the rapid progression following disease recurrence.

To test this hypothesis, we isolated Taxol-resistant cell lines from parental ovarian cancer cell lines SKOV3 and OVCAR3 using a metronomic approach by repeated exposure to clinically relevant concentrations of Taxol^12^ with intermediate recovery periods similar to therapeutic administration. This produced IC_50_ values of 20–45 nM, equivalent to the latter studies that observed alterations in microtubule dynamics^22^,^23^. Initial studies suggested that these changes were not due exclusively to changes in drug efflux or other direct pro-survival adaptions but Taxol-resistant cells did show increased microtubule dynamics, including increased microtubule growth rates and decreased levels of polymerized tubulin. Analysis of attachment kinetics revealed that Taxol resistant cells adhered nearly two-fold faster, which correlated with higher integrin expression. In contrast, analysis of adhesion strength using a centrifugal-force based adhesion assay revealed Taxol-resistant cells attached less strongly to the ECM. To understand the decreased adhesion strength despite increased attachment rate and integrin expression we stained for intracellular focal adhesions and found that Taxol resistance dramatically reduced both their size and number. Finally, we sought to determine if these changes in adhesion and microtubule polymerization occurred independently or if they were causally related. Though chemical perturbation of microtubule polymerization did not alter adhesion strength, inhibition of focal adhesion kinase (FAK) mitigated adhesive differences between parental and Taxol-resistant cells. Additionally, microtubule dynamics were also suppressed following FAK inhibition in both cell lines producing statistically identical growth rates in both the parent and Taxol-resistant cells. These results highlight a novel mechanism of ovarian cancer chemoresistance, and may provide therapeutic targets such as focal adhesion kinase to both slow metastatic cell engraftment and increase chemosensitivity.

## Results

### Taxol resistance is independent of drug efflux and does not confer additional chemoresistance

**Figure 1:**
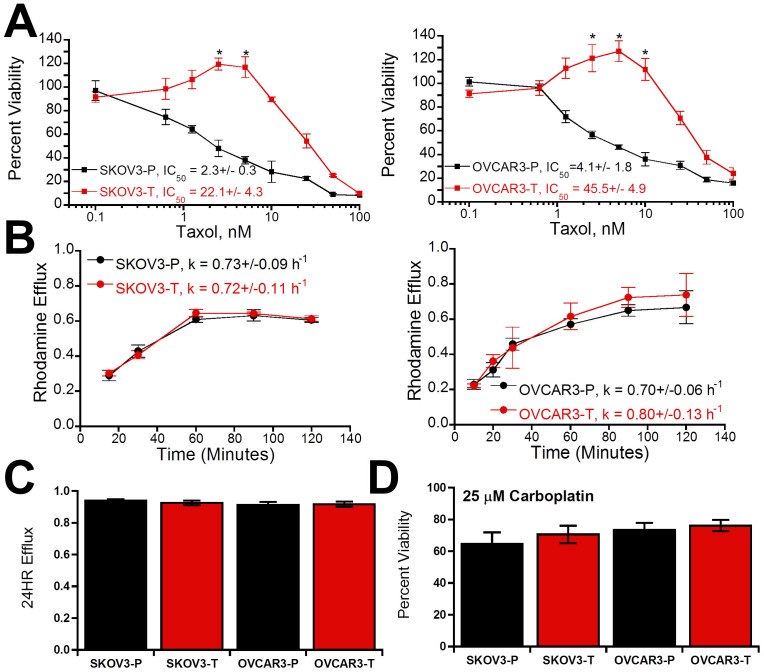
Generation of Taxol-resistant populations and analysis of potential resistance mechanisms. (A) Viability of parental (-P) and Taxol-resistant (-T) populations of SKOV3 and OVCAR3 ovarian cancer cells in after incubation in varying concentrations of Taxol (* is significantly greater than solvent treated control, p < 0.05, N = 3). (B) Time-dependent rhodamine efflux over initial two hours was used to calculate efflux rates (k), which showed no significant difference among cell populations (N = 3). (C) Long-term 24 hour efflux showed no significant difference between cell populations (N = 3). (D) Viability after incubation with 25 μM Carboplatin relative to solvent-treated control. Values given as mean ± SEM.

After establishing populations of SKOV3 and OVCAR3 ovarian cancer cell lines capable of growing in Taxol, we first verified this correlated with an increase in IC_50_ by treating cells with Taxol at varying concentrations for 72 hours. Consistent with previous reports^25^, SKOV3 and OVCAR3 parental populations (abbreviated as SKOV3-P and OVCAR3-P) showed IC_50_ values of 2.3 ± 0.3 nM and 4.1 ± 1.8 nM, respectively, which were increased an order of magnitude in the Taxol-resistant subpopulations (abbreviated as SKOV3-T and OVCAR3-T) to 22.1 ± 3.0 nM and 45.5 ± 4.9 nM (Fig. 1A). While parent populations show dose-dependent decreases in viability, Taxol-resistant cells showed significantly increased viability at concentrations of 5–10 nM Taxol beyond which viability decreased. To begin to investigate the mechanism of this resistance, we next evaluated the ability of the cells to efflux Rhodamine 123 as a model drug (Fig. 1B–C), as both Taxol and Rhodamine 123 are substrates for P-glycoprotein mediated efflux^26^. Evaluation of both efflux kinetics (Fig. 1B) and total efflux after twenty four hours (Fig. 1C) demonstrated no significant changes with Taxol resistance. Finally, to see if changes were the product of other pro-survival adaptions we assayed the ability of cells to survive in 25 μM Carboplatin and found no significant change in Carboplatin resistance in Taxol resistant subpopulations (Fig. 1D). These results suggest that alternative mechanisms must be underlying this Taxol resistance.

### Microtubule alterations in Taxol-resistant cells

**Figure 2:**
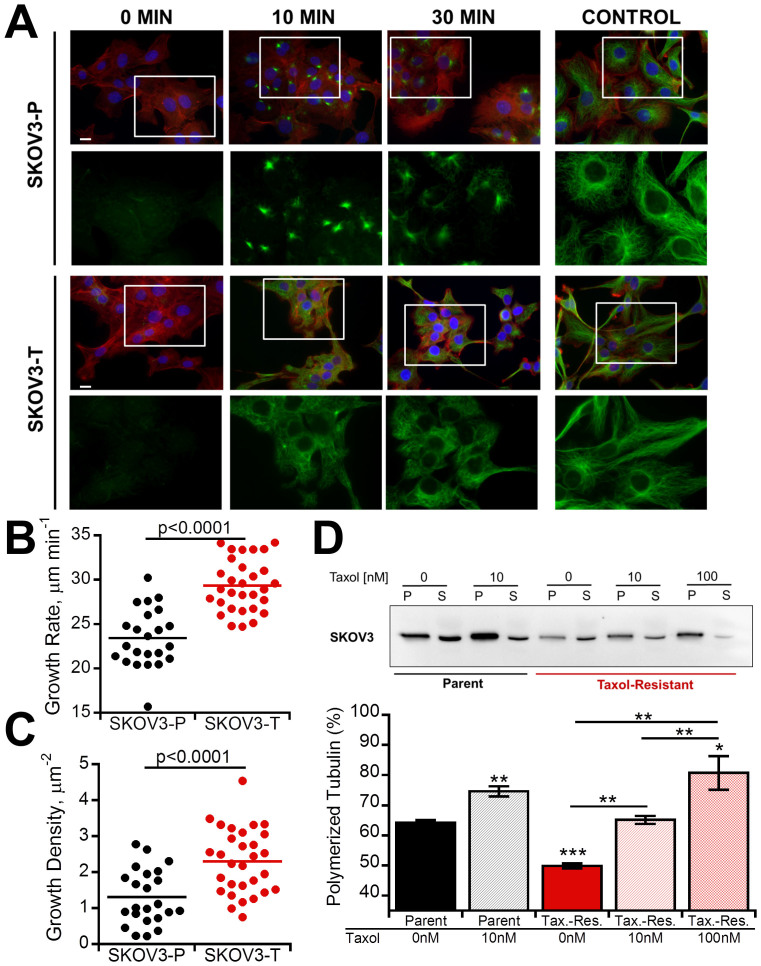
Microtubule dynamics are increased in Taxol-resistant cells. (A) Microtubules were depolymerized with nocodazole for four hours and then drug was washed out for indicated time at which point cells were immunostained for microtubules (green), actin (red), and nuclei (blue). Scale bar = 10 μm. (B–C) Live-cell microtubule dynamics as determined from plus-end tracking of fluorescent EB3 (see Videos 1 and 2). Following transfection with mCherry-EB3, growing microtubule plus-ends were imaged at 1 Hz for 2 minutes to determine growth rate (B) as well as growth density (C), defined as the total number of growing ends normalized to cell area. Each dot represents the average of over 100 tracked plus-ends from one cell collected from a total of 4 independent experiments. (D) Taxol-resistant cells have less polymerized tubulin. Cells were lysed following 4 hour pretreatment with Taxol and separated into polymerized (P) and soluble (S) tubulin fractions for Western blot analysis. Percent polymerized tubulin was quantified as polymerized tubulin divided by the sum of polymerized and soluble tubulin. A cropped representative blot from 4 independent experiments is shown, and the full-length blot is available in the supplemental information (Fig. S6). Values given as mean ± SEM; significance is indicated relative to control parent population unless otherwise noted, *P < 0.05, **P < 0.01,***P < 0.001.

Based on the increase in viability seen with low-dose Taxol in Taxol-resistant cells, we next evaluated if Taxol-resistant cells displayed enhanced microtubule dynamics. First, we performed a microtubule regrowth assay where microtubules were depolymerized with nocodazole and then allowed to regrow following washout (Fig. 2A). By 10 minutes SKOV3-P cells had begun to nucleate whereas microtubule networks had already begun to form in SKOV3-T cells, which was not seen until 30 minutes in the SKOV3-P cells. To verify this difference in live cells in absence of chemical perturbation we transfected cells with fluorescent end-binding protein (mApple-EB3) which binds to the growing plus ends of microtubules allowing for quantification of microtubule growth rates (Supp. Vid. 1–2)^27^. Not only were microtubule growth rates significantly faster in the SKOV3-T cells (p < 0.0001, Fig. 2B), but they also showed an increased number of growing plus ends (p < 0.0001, Fig. 2C), indicating significantly increased dynamics. Finally, we performed a microtubule pelleting assay to determine if the cells natively had different levels of polymerized microtubules revealing SKOV3-P cells had significantly more polymerized tubulin than SKOV3-T cells (Fig. 2D). Treating SKOV3-T with 10 nM Taxol returned levels of polymerized tubulin to those of parental cells, while 100 nM Taxol was required to increase tubulin polymerization above parent levels. With similar findings in OVCAR3-T cells (Fig. S1), these results indicate that Taxol-resistant cells have decreased polymerized microtubules and increased microtubule growth rates consistent with previous reports^9^,^10^,^11^.

### Increased attachment kinetics correlate with integrin expression in Taxol-resistant cells

**Figure 3:**
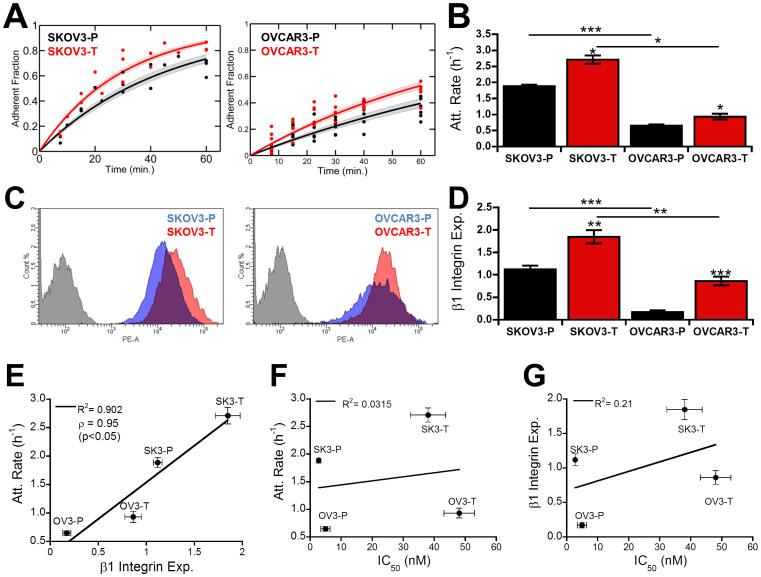
Taxol resistance alters attachment kinetics through β1 integrin. (A) Cells fluorescently labeled green with Calcein AM were plated for specified period of time before removing non-adherent cells and quantifying the adherent fraction fluorometrically. Individual dots represent independent experiments; solid lines are fit curves with shaded region representing the 95% confidence interval. (B) Attachment rate determined from regression of each independent experiment. (C–D) Representative flow cytometry intensity histograms for cells labeled with PE-CD29 (β1 integrin) (C) as well as mean fluorescence intensity of surface β1 integrin (D), normalized to the mean of each individual experiment to account for any variations in laser intensity (N = 3). (E) Surface expression of β1 integrin shows direct linear correlation with adhesion rate (Pearson correlation coefficient ρ = 0.95, R^2^ = 0.902). (F–G) Neither β1 integrin (F) or adhesion rate (G) correlated with Taxol sensitivity. Values given as mean ± SEM; significance is indicated relative to control parent population unless otherwise noted, *P < 0.05, **P < 0.01,***P < 0.001.

Several recent studies have shown a strong link between microtubules and focal adhesions^22^,^28^,^29^. Thus, we sought to determine if changes in Taxol sensitivity produced alterations in cell adhesion. To do so, we first analyzed the attachment kinetics as cells initially attached to a collagen-coated surface (Fig. 3A). Though SKOV3 cells adhered faster overall, the Taxol-resistant populations both adhered faster than their parental counterparts (Fig. 3B). This increase in attachment rate correlated with increased spreading (Fig. S2, Fig. S3A) and was conserved on both fibronectin and Matrigel coated surfaces (Fig. S3B). Due to previous studies linking increased integrin expression and chemoresistance^17^. we hypothesized the increased attachment rate in Taxol-resistant cells may be due to integrin overexpression. Surface integrin expression was quantified using flow cytometry for β1 integrin responsible for binding to collagen I (Fig. 3C). For both cell lines, surface integrin expression was increased in Taxol-resistant clones, though SKOV3 cells expressed higher overall levels of β1 integrin (Fig. 3D). These results directly correlated with observed attachment kinetics, showing a positive linear relationship between β1 integrin expression and attachment rate (R^2^ = .90, Pearson's correlation coefficient ρ = 0.95, Fig. 3E). However, due to basal variations between SKOV3 and OVCAR3 neither attachment rate (R^2^ = 0.03, Fig. 3F) nor integrin expression (R^2^ = 0.21, Fig. 3G) correlated with IC50, suggesting though this may be a contributing factor it was not a primary mechanism of Taxol resistance.

### Taxol resistance decreases adhesion strength through diminished focal adhesion formation

**Figure 4:**
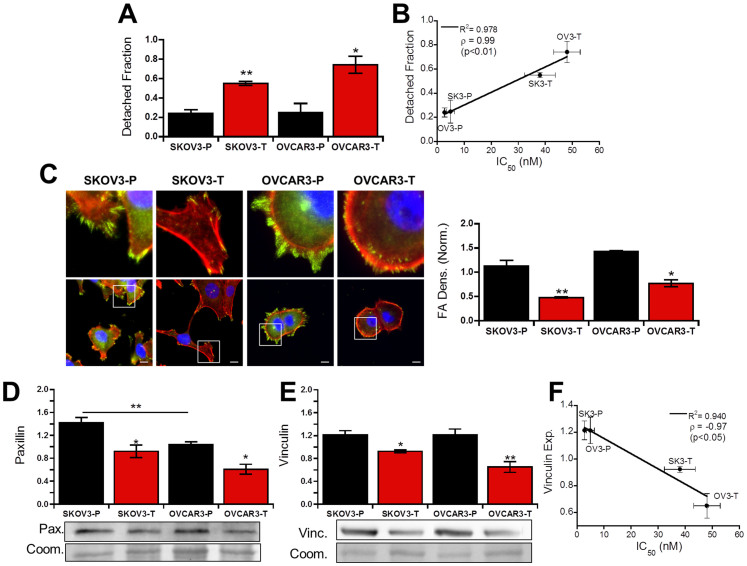
Taxol resistance decreases adhesion strength by altering focal adhesions. (A) Detached fraction of parent (-P) and Taxol-resistant (-T) cells allowed to adhere overnight before detachment by centrifugal force shows significantly decreased adhesion strength in Taxol-resistant clones (p < 0.01,N = 4). (B) Detached fraction of cells correlates directly with IC50 values (p < 0.01), Pearson correlation coefficient ρ = 0.99. (C) Immunofluorescent micrographs of cells labeled for paxillin (green), F-actin (red), and nuclei (blue) with zoomed versions of highlighted areas. Scale bar = 10 μm. Focal adhesion density was quantified as the integrated density of segmented focal adhesions relative to cell area (N = 3). (D–E) Total paxillin (D, N = 4) and vinculin (E, N = 3) expression, quantified by Western blot normalized to total protein, is decreased in Taxol-resistant cells but is also dependent on cell line. A cropped representative blot is shown, and full-length blots are available in the supplemental information (Fig. S6). For total protein from Coomassie, a representative region of the quantified area is shown, complete image is available in Figure S6. (F) Vinculin expression inversely correlates with IC50 values (p < 0.05), Pearson correlation coefficient ρ = −0.97.

The increased integrin expression suggested that adhesion strength would be increased in Taxol-resistant cells, but inhibition of tubulin polymerization has previously been shown to effect focal adhesion formation and steady state adhesion strength^30^,^31^. Since Taxol-resistant cells displayed decreased polymerized tubulin, we next quantified their adhesion strength using a centrifugal-force based adhesion assay to test if the integrin up-regulation in Taxol-resistant clones led to increased adhesion strength. After adhering overnight, the detached fraction following centrifugation in Taxol-resistant population was nearly twice that of their parental lines (Fig. 4A), despite integrin overexpression. This result correlated well with changes in IC_50_ (Fig. 4B) suggesting alterations in Taxol sensitivity may be related to alterations in adhesion strength.

Based on this, we hypothesized that Taxol resistance was decreasing focal adhesion formation; therefore, focal adhesions were visualized by staining for the focal adhesion protein paxillin (Fig. 4C). Cells from both parent populations contained large focal adhesions distributed throughout the cell periphery. In the Taxol-resistant populations these focal adhesions were smaller and more disperse. Quantification of paxillin localized to focal adhesions showed significant reductions in both Taxol-resistant cell lines compared to their respective parent lines (Fig. 4C). Similar results for the focal adhesion protein vinculin were observed in OVCAR3 cells; however neither population of SKOV3 showed significant vinculin positive focal adhesion expression with only disperse staining (Fig. S4A). Analysis of total paxillin expression by Western blot showed decreased expression in Taxol-resistant cells (Fig. 4D). Paxillin levels were equivalent between SKOV3-T and OVCAR3-P even though the latter exhibits significantly higher adhesion strength (Fig. 4A), indicating the decreased adhesion strength is not solely due to decreased paxillin expression. Surprisingly, despite the lack of vinculin-containing adhesions in SKOV3 cells, expression of vinculin agreed best with adhesion strength results (Fig. 4E) and also inversely correlated with Taxol sensitivity (Fig. 4F).

While total vinculin expression does seem to agree with adhesion strength, the disparate distribution of vinculin between SKOV3 and OVCAR3 cells suggests alternative molecules may be a larger determining factor. Since vinculin has been shown to inhibit paxillin interactions with focal adhesion kinase (FAK)^32^, we also stained for FAK phosphorylated at Y397 (FAKp397). In parental cells FAKp397-positive adhesions tended to be larger but less numerous than paxillin-positive focal adhesions, whereas in Taxol-resistant cells FAKp397-positive adhesions tended to appear as more nascent adhesions both smaller in size and more disperse (Fig. S4B). Taxol-resistant cells also expressed higher overall levels of FAKp397 by Western blot (Fig. S4C). Consistent with the idea that parental cells tend to have larger more mature adhesions whereas Taxol-resistant cells tend to have more nascent adhesions which are known to exert larger forces^33^, traction force cytometry revealed that Taxol-resistant cells exert nearly 2-fold larger forces than their parental counter parts (Fig. S5). These results demonstrate that the decreased adhesion strength coincides with smaller nascent focal adhesions which exert higher forces as a result of changes in expression of adhesion proteins. These changes agree with decreased vinculin expression in Taxol-resistant cells, but do not correlate with paxillin expression or integrin expression suggesting additional intracellular signaling cascades contribute to the decreased adhesion strength.

### Alterations in adhesion strength are dependent on focal adhesion kinase and upstream of microtubule dynamics

**Figure 5:**
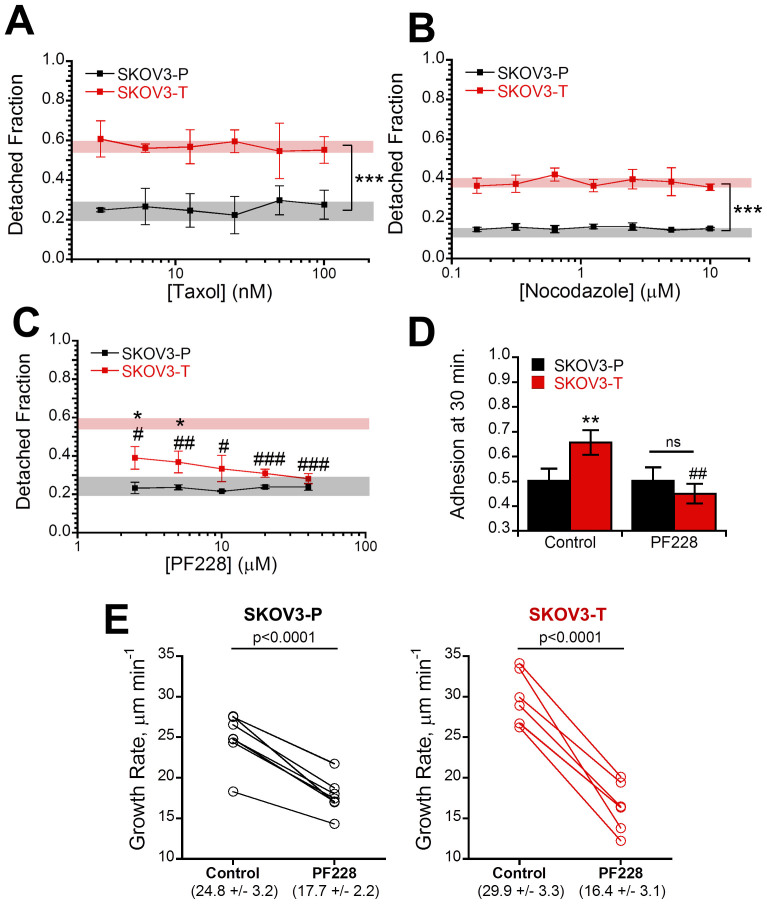
Alterations in adhesion and microtubule dynamics in Taxol-resistant cells are reversible with FAK inhibition. (A) SKOV3 parent (-P) and Taxol-resistant (-T) were pre-incubated with 1:1 serial dilutions ranging of Taxol ranging from 3.13–100 nM for 4 hours to stabilize microtubules before repeating the adhesion strength assay and displayed no change from the untreated controls (shaded region) (N = 3). (B) The adhesion strength assay was repeated using 4 hour nocodazole pre-treatment with 1:1 serial dilutions ranging from 0.16–10 μM to depolymerize microtubules (N = 3). In order to prevent changes from increased Rho activity upon nocodazole upon washout the experiment was carried out in the drug-containing media instead, producing a slightly higher baseline for SKOV3-T. (C) To inhibit focal adhesion signaling cells were pre-incubated with 1:1 serial dilutions of focal adhesion kinase inhibitor PF228 ranging from 2.5–40 μM for 4 hours before running the adhesion strength assay demonstrating selective recovery of adhesion force in Taxol-resistant cells relative to their untreated controls (red shaded region) to become equivalent with parent control cells (gray shaded region) (N = 3). (D) To determine the effects of FAK inhibition on attachment kinetics, cells were pre-incubated with 10 μM PF228 for 30 minutes in solution and allowed to adhere for 30 minutes revealing FAK inhibition decreased attachment kinetics in Taxol-resistant cells (N = 3). (E) Cells transfected with mCherry-EB3 were imaged and then treated with 10 μM PF228 for four hours before re-imaging revealing a significant (p < 0.0001) decrease in both parent and Taxol-resistant cells to a statistically equal value. Values listed in parenthesis are given as mean ± std. All other values given as mean ± SEM; significance is indicated relative to matched parent population with *′s and relative to solvent treated control #′s. *P < 0.05, **P < 0.01,***P < 0.001.

We next sought to determine what intracellular signaling cascades could be responsible for the alterations in adhesion strength and microtubule dynamics. Based on our observations, we hypothesized that either (1) alterations in microtubule dynamics were decreasing focal adhesion formation or (2) decreased focal adhesion formation was altering microtubule dynamics. To determine which mechanism was ultimately altering the adhesive strength of Taxol-resistant cells we chemically modified both pathways and repeated the adhesion strength assay. First, to test if the decreased adhesion strength was due to increased microtubule dynamics we pre-incubated cells with either Taxol to stabilize (Fig. 5A) microtubules or nocodazole to depolymerize microtubules (Fig. 5B) and repeated the adhesion strength assay. In both cases, there was no change in the adhesive strength of either the parent or Taxol-resistant cells across a wide array of concentrations indicating the changes in adhesion strength are not due to microtubule dynamics. To investigate if these changes were due to altered focal adhesion signaling we inhibited the increased FAK phosphorylation observed in Taxol-resistant cells with PF228 (Fig. S4B–C), which has been shown to block focal adhesion turnover^34^. In parental cells FAK inhibition did not alter adhesion strength at any tested concentration (Fig. 5C, black line). In contrast to this, the detached fraction was significantly lower than untreated control for all concentrations in Taxol-resistant cells and no longer significantly different than the parental cell line for all concentrations greater than 5 μM (Fig. 5C, red line). To probe if FAK inhibition could also reverse changes in attachment kinetics, we allowed cells to adhere for 30 minutes after pretreatment with PF228 and found FAK inhibition significantly reduced Taxol-resistant cell attachment with equal fractions of both parental and Taxol-resistant cells adhering in this short time scale (Fig. 5D). Finally, to verify that changes in adhesive signaling were the up-stream cause we repeated the microtubule plus-tip tracking with EB3 after FAK inhibition and found a significant (p < 0.001) decrease in microtubule growth rate for both SKOV3-P and SKOV3-T cells (Fig. 5E). Notably, this produced equivalent growth rates in both cell populations through a larger growth rate decrease in the Taxol-resistant cells (45% vs. 28%). Taken together, these results suggest a model where changes in focal adhesion signaling cause alterations in microtubule dynamics leading to increased resistance to microtubule-stabilizing drugs such as Taxol.

## Discussion

Taxol is clinically effective in treating a variety of cancers, including breast and ovarian cancer, but eventual acquired resistance limits its long-term efficacy. Some studies suggest this may be through increased drug efflux or altered survival signals^5^,^8^. For instance, early evidence showed Taxol resistance can cause over expression of the P-glycoprotein (Pgp) drug efflux pump^7^. Indeed, highly Taxol resistant populations of ovarian cancer show increased expression of multiple drug resistance pumps^35^. Despite this extremely promising *in vitro* data, clinical trials of drug pump inhibitors have failed to meet general clinical end points^36^. High early Pgp expression is an indicator of poor prognosis; therefore targeting of these patients with high de novo expression may yield more positive results^37^.

One potential reason for the lack of success of drug pump inhibitors is many studies on chemoresistance were conducted in populations resistant to concentrations much greater than occur therapeutically^12^. Resistance to such high concentrations may activate alternative chemoresistance mechanisms that would not be relevant in the clinic. Moreover, isolation of Taxol-resistant ovarian cancer cells with high initial Taxol concentrations confers cross-resistance to other chemotherapeutics that is minimized when isolated with initially lower concentrations^38^. The Taxol-resistant cells isolated here using our metronomic approach of repeated exposure and withdrawal of clinically relevant concentrations of Taxol^12^ produced lines with IC50 values of 20–50 nM. These Taxol resistant populations that showed no difference in ability to efflux Rhodamine 123 (Fig. 1B–C), which is also effluxed by the multidrug transporter P-glycoprotein responsible for Taxol efflux^26^. Moreover, the lack of change in resistance to carboplatin suggests the resistance is not due to either increased survival signals or decreased sensitivity to death signals (Fig. 1D). Another potential mechanism lies in modifications to microtubule isoforms and dynamics^39^. Several of these studies, like ours, used less resistant clones with IC_50_ values in the 20–40 nM range^9^,^10^,^11^. In agreement with their findings, we found decreased levels of polymerized tubulin and increased microtubule dynamics within our Taxol-resistant cells (Fig. 2). Treatment of Taxol-resistant cells with 10 nM Taxol returned level of polymerized tubulin to those of untreated parental populations (Fig. 2C, Fig. S1). This result may explain the increased viability in Taxol-resistant cells that peaks at similar Taxol concentrations (Fig. 1A), which could act to stabilize the hyper-dynamic microtubules in Taxol-resistant cells to normal levels. In the absence of Taxol, the resistant cells' microtubules may not have the stability necessary to properly form the mitotic spindle for division, which is then recovered by low-dose Taxol.

Since microtubules have been shown to regulate focal adhesion assembly and disassembly^24^ and focal adhesions have also been shown to alter microtubule dynamics^22^,^23^, we hypothesized that Taxol resistance may result in altered cell adhesion. While both attachment kinetics (Fig. 3A–B, Fig. S3B) and strength (Fig. 4A, Fig. S3C) were altered, only adhesion strength correlated with Taxol sensitivity (Fig. 3F, Fig. 4B), whereas attachment kinetics were more strongly correlated with integrin expression (Fig. 3E). As expected, this decreased adhesion strength coincided with smaller focal adhesions in Taxol-resistant cells (Fig. 4C, Fig. S4). Early studies into the effects of microtubules on focal adhesions demonstrated that microtubules are critical for focal adhesion disassembly and their stabilization with Taxol results in increased focal adhesion size^40^. Consistent with this, parental cells with more polymerized and less dynamic microtubules (Fig. 2) displayed larger focal adhesions (Fig. 4C, Fig. S4). Recent studies have also shown focal adhesion dissolution may be facilitated by CLASP proteins which bind microtubule plus-ends to adhesion sites where they secrete MMPs to degrade the underlying matrix expediting focal adhesion turnover^41^. The higher density of microtubule plus-ends in Taxol-resistant cells (Fig. 2C) may thus expedite focal adhesion turnover leading to less large adhesions.

In another classic work, depolymerization of microtubules with nocodazole in serum-starved fibroblasts was shown to increase formation of focal adhesions, which then dissolve and turn over following drug washout as microtubules regrow^28^. In this model, though FAK null and FAK expressing fibroblasts formed focal adhesions during depolymerization to a similar degree, only cells expressing FAK were able to dissolve focal adhesions following microtubule regrowth. This microtubule depolymerization also corresponds to increased traction forces, which occurred in absence of FAK or myosin II activation, but was blocked by simultaneous inhibition of both^29^. Here, we likewise saw larger traction forces in Taxol-resistant cells with more dynamic microtubules (Fig. S5). However, consistent with previous reports^42^ we found no difference in adhesion strength upon stabilizing (Fig. 5A) or depolymerizing microtubules (Fig. 5B) suggesting an alternative microtubule-independent signaling pathway was controlling the phenomena.

Focal adhesion turnover is FAK-dependent^28^,^34^, so we next inhibited FAK with PF228 to attempt to recover adhesion strength. We found it selectively increased adhesive strength in Taxol-resistant cells (Fig. 5C) and likewise recovered differences in attachment kinetics (Fig. 5D). Similar trends were found by Michael et. al. using FAK-null fibroblasts expressing tetracycline-regulated FAK where FAK expression increased adhesion kinetic parameters but decreased steady-state strength^43^. Finally, we verify that FAK is acting up-stream of changes in microtubule dynamics by tracking microtubule plus-ends before and after FAK inhibition. We found significantly decreased microtubule growth rates in both parent and Taxol-resistant populations (Fig. 5D) with statistically equal growth rates in both treated populations (p = 0.37). Conversely, others have shown that active FAK acts via Rho signaling to facilitate the formation stable Glu-microtubules from the more dynamic tyrosinated microtubules^22^. This suggests that FAK inhibition should increase microtubule dynamics; however, these studies in fixed cells may not be able to fully capture microtubule dynamics. Other researchers have noted similar differences when comparing live-cell microtubule dynamics to results quantified from fixed cells stained for Glu-microtubules^44^. One potential explanation is that ovarian cancer cells, which are known to overexpress Rho^45^, have sufficient basal Rho signaling to circumvent the need for FAK activity to form stabilized microtubules. As noted by Salaycik and colleagues, this may also be because stabilized Glu-microtubules only represent a relatively small fraction of microtubules within the cell, primarily localized to the perinuclear region and not the lamella, or that these signals are required for the generation, but not maintenance of, Glu-microtubules^44^. The increased formation of stable Glu-microtubules also does not preclude the remaining majority of microtubules from exhibiting increases in dynamics.

The hypothesis that FAK intracellular signaling pathway is altering adhesion formation is further supported by the increase in β1 integrin expression in Taxol-resistant populations (Fig. 3D). We observed decreased adhesion strength despite higher levels of integrin expression (Fig. 4A). Though this result is somewhat counterintuitive, focal adhesion strengthening is largely dependent on both integrin clustering and focal adhesion formation, but can occur without additional binding of integrins to the extracellular matrix^46^. Modeling of focal adhesion bond strength indicates that focal adhesion assembly alone can double adhesion strength in absence of integrin binding or clustering^47^, in good agreement with our observations. Additionally, the collagen I receptor integrin α2β1 is known to activate FAK, so increased β1 expression may lead to higher FAK activity promoting focal adhesion dissolution^48^. There may be additional defects in focal adhesion formation/maturation due to vinculin down-regulation in Taxol-resistant cells (Fig. 4E). Loss of vinculin has been shown to increase paxillin-FAK interactions leading to higher phosphorylation of both^32^, causing increased focal adhesion disassembly rates^49^.

This finding adds to a body of work suggesting that FAK inhibition may be an effective treatment for advanced ovarian cancer^50^,^51^,^52^. Previous work has demonstrated that decreasing FAK activity through either FAK silencing^50^ or inhibition^51^ increases ovarian cancer sensitivity to taxanes. This suggests a causal role for focal adhesion dynamics in Taxol resistance. In addition to altering microtubule dynamics as shown here, FAK has also been shown to act through additional pathways such as YB1^51^. Furthermore, FAK is significantly up-regulated in ovarian cancer (p = 1.71e-5), more than any other tumor site^53^ and FAK overexpression has been linked to poor prognosis with an over two-fold decrease in median survival^52^. Completed phase I trials of FAK inhibitors in multiple solid tumors show promise^54^, and phase I/Ib trials of FAK inhibitors with Taxol are currently underway in patients with advanced ovarian cancer (NCT01778803).

In addition to changes in chemoresistance, these changes in adhesion dynamics may also contribute to cancer metastasis, which is ultimately responsible for 90% of cancer deaths^13^. In order to metastasize, cells must first escape the primary tumor. Decreases in focal adhesion strength could increase the frequency of this event, making it easier for cells to begin spreading to distant sites. Moreover, during this process focal adhesion turnover is critical for effective migration; if focal adhesions grow too large the excessive adhesion prevents cell translocation^55^. In ovarian cancer, altering these processes by blocking FAK inhibits tumor cell migration and invasion^52^. Once reaching the secondary site, the faster attachment kinetics from increased integrin expression could increase the rate at which cells engraft. The interaction of mesothelial VCAM-1 with its ligand α4β1 integrin is critical for mesothelial cell clearance and ovarian cancer metastasis^20^. This process additionally requires cell-generated traction forces found to be up-regulated in Taxol-resistant cells (Fig. S5)^56^. These findings indicate blocking integrin signaling may also be a successful adjuvant therapeutic in ovarian cancer. Early clinical trials of anti-integrin therapeutics have already shown success in glioblastoma with minimal toxicity^17^, and promise with ovarian cancer as well^57^. Several studies also suggest increased integrin expression can lead to general chemoresistance^58^ and is a marker of poor patient prognosis^59^. The integrin up-regulation may also act synergistically with FAK, as integrin-dependent FAK activation from the ascites can also protect ovarian cancer cells from death by Akt phosphorylation^60^.

In conclusion, this study demonstrates previously unknown changes in adhesion properties of cells resistant to chemotherapeutics. These alterations correlated with an increase in microtubule dynamics, but were unaffected by chemical perturbation of microtubule dynamics. FAK inhibition not only mitigated adhesive differences between parent and Taxol-resistant cells, but also normalized microtubule dynamics. Thus, FAK could potentially be therapeutically targeted to not only increase chemosensitivity, but also block metastasis to improve the extremely poor prognosis of chemorefractory ovarian cancer.

## Methods

### Cell culture and isolation of Taxol-resistant cells

Human ovarian carcinoma SKOV-3 cells were acquired from ATCC and OVCAR-3 cells were the generous gift of Dr. John McDonald. Both cell lines were cultured in RPMI 1640 (Corning) containing 10% FBS (Atlanta Biologicals) and 1% penicillin streptomycin (Corning). To isolate Taxol resistant cell lines, cells were plated at 20% of confluence and treated with 10 nM Taxol (Enzo) for 48 hours before returning to growth media. After reaching 40% of confluence, cells were again treated with 10 nM Taxol for 48 hours before returning to growth media. This process was repeated until the Taxol could no longer maintain cell growth (approximately 4 months for OVCAR3 and 6 months for SKOV3), at which time cells were fed with standard growth media with a maintenance concentration of 5 nM Taxol added at least once per week. Four independent viability assays were performed over the course of the study with no significant change in IC_50_.

### Viability assay

Cells were plated at 20% of confluence before treating with varying concentrations of Taxol, 25 μM carboplatin (approximately the IC_50_ for both cell lines)^61^, or a DMSO solvent control (<0.1% v/v). After 72 hours, cells were incubated in 1 mg/mL MTT reagent for four hours in standard growth conditions. The supernatant was then removed and replaced with isopropanol acidified with 4 mM HCl to solubilize insoluble purple formazan product. The absorbance of this product was then measured in DTX-800 Multimode Detector microwell plate reader (Beckman Coulter) at 620 nm absorbance with 595 nm reference value. Since low-dose Taxol showed a significant increase in Taxol-resistant cell viability we chose to interpolate the IC_50_ in the linear region of the plot instead of a standard sigmoidal fit which may not accurately model the data.

### Attachment kinetics

All plates were coated with 10 μg/mL Collagen I (Corning) and blocked with 1% heat-denatured BSA (Rockland) unless otherwise noted. Cells were first labeled with 2 μM Calcein AM (AnaSpec), a transmembrane green fluorescent marker, in HBSS with divalents (Corning) for 20 minutes at 37°C. Next, cells were passed per standard procedure, resuspended in media, and incubated for 30 minutes at 37°C. Cells were then pelleted and resupsended in adhesion buffer (140 mM NaCl, 2.5 mM KCl, 1.8 mM CaCl_2_, 1.0 mM MgCl_2_, 20 mM HEPES, 20 mM dextrose, pH 7.4) before plating at 20% of confluence. At each time point, non-adherent cells were removed to a new plate. At the end of the experiment, the fluorescence of the plates containing both the adherent and non-adherent fractions was read at 485 nm excitation, 535 nm emission in a DTX-800 Multimode Detector microwell plate reader. Adherent fraction was then defined as the reading of the adherent cells over the sum of the adherent cells and non-adherent cells. Attachment was modeled by the differential equation *dC_Adh_*/*dt* = *kC_Non_*_-*adh*_ where C is the concentration of cells and k is the adhesion rate. This equation can be reduced to *dA*/*dt* = *k*(1 − *A*) where A is adherent fraction calculated as *A* = *C_Adh_*/(*C_Adh_* + *C_Non_*_-*adh*_) and solved using boundaries of A = 0 and A = 1 at t = 0 and as t → ∞, respectively, to yield *A*(*t*) = 1 − *e*^−*kt*^. The equation was linearized, yielding R^2^ values for individual experiments ranging from 0.92 to 0.99.

### Adhesion strength

Adhesion strength was quantified in a centrifugal force-based adhesion assay^62^. Cells were allowed to adhere overnight to a collagen-coated (unless otherwise noted) 96 well plate and then labeled with Calcein AM. The media was replaced with adhesion buffer before taking an initial fluorescence reading, and then plates were inverted and centrifuged at 29 rcf for 5 minutes. After washing with adhesion buffer, a final reading was taken. Detached fraction was determined as one minus final fluorescence divided by initial fluorescence. In some experiments, cells were pre-incubated with Taxol, Nocodazole (Sigma), or PF-573,228 (PF228, Sigma) 4 hours before performing the experiment.

### Rhodamine efflux

Cells in a 96 well plate were loaded with 0.5 μg/mL Rhodamine 123 for 60 minutes and then washed extensively with adhesion buffer. At desired time points, supernatant was removed for fluorescence quantification at 485 nm excitation, 535 nm emission. To account for variations in loading, rhodamine efflux was determined as [effluxed rhodamine/(effluxed rhodamine + rhodamine in cells)]. Rate constants were fit as described in attachment kinetics. For 24 hour efflux, a similar procedure was performed except cells were incubated in growth media.

### Flow cytometry

To determine levels of surface integrin expression, cells were analyzed with a BD LSR-II flow cytometer. Briefly, cells were detached with 5 mM EDTA, centrifuged, and separated into 100 μl aliquots then labeled with PE-conjugated anti-human CD29 (integrin β1, Clone TS-2/16, BioLegend) per manufacturer's instructions. Mean fluorescence intensity was determined after subtraction of a respective negative control.

### Focal adhesion staining and image analysis

For immunostaining all cells were plated on collagen I coated coverslips. For focal adhesion staining, cells were fixed in 4% formaldehyde, permeabilized with 0.5% Triton X-100, and blocked with 5% horse serum before staining with either 1:200 anti-paxillin (Clone Y113, GeneTex), 1:500 anti-vinculin (Invitrogen), or 1:200 anti-FAKp397 (Genetex) diluted in PBS with 1% BSA. Cells were then washed, incubated with 1:100 rhodamine phalloidin (Invitrogen) and anti-rabbit Alexa Fluor 488 (Invitrogen), counterstained with DAPI (AnaSpec), and sealed with Vectashield (Vector Labs). All images were captured at 40× magnification on an inverted Nikon Microscope with a CoolSNAP camera (Photometrics). For each experiment, all images were captured in one session and normalized to the average for that session to account for any differences in light brightness. To quantify focal adhesions, paxillin images were first convolved with a low pass Gaussian filter before applying a morphological top hat filter to correct for differences in basal paxillin expression. Pixels within the cell area (determined based on F-actin fluorescence) that exceeded the cell background by 2 standard deviations were considered positive. After thresholding, pixel noise of less than 10 pixels (~0.25 μm^2^) was discarded. The remaining segmented focal adhesions were used for analysis, with pixel intensity values taken from the original, unfiltered image after background subtraction (background defined as the average intensity value of the non-cell area). Focal adhesion density was calculated as the integrated focal adhesion intensity normalized to cell area.

### Western blot analysis

Cells were lysed in radioimmunoprecipation buffer containing a protease inhibitor cocktail and separated on either 10% (paxillin/tubulin) or 7.5% (vinculin/FAKp397) polyacrylamide gel before transferring to a PVDF membrane. Membranes were blocked in 5% milk, and incubated overnight at 4°C in primary antibodies against tubulin (1:3000, Rockland), paxillin (1:500, BioLegend), vinculin (1:1000, Invitrogen), or FAKp397 (1:1000, Genetex). Bands were visualized following incubation with 1:1000 HRP-conjugated anti-rabbit IgG (Rockland) using Novex ECL chemiluminescent substrate (Invitrogen). Since typical loading controls such as cytoskeletal elements (tubulin/actin) or metabolic enzymes (GAPDH) are often differentially expressed in cancer, we utilized total protein as a loading control by staining the membranes with Coomassie G250 (BioRad). Quantification of total protein has been not only been shown to be more consistent across different cells than typical loading control proteins, but also offer improved detection linearity^63^,^64^. All analysis was performed using built-in blot analysis tools in ImageJ (NIH) as described in the user manual (http://rsbweb.nih.gov/ij/docs/guide/, Section 30.13). Total protein was quantified as the integrated density of the lane in the area indicated in Figure S6.

### Tubulin polymerization assay

A microtubule pelleting assay was used to quantify fraction of polymerized tubulin as previously described^65^. Cells that had been in absence of Taxol for 5–7 days were grown to 80% confluence in a 24-well plate. Cells were lysed with 100 μL hypotonic lysis buffer (20 mM Tris-HC1, pH 6.8, 0.5% Nonidet P-40, 1 mM MgC12, 2 mM EGTA) for 5 minutes. Lysates were transferred to a 1.5 mL microcentrifuge tube, wells were washed 1x with 100 μL hypotonic lysis buffer, and the entire 200 μL volume was vortexed before pelleting the insoluble fraction by room temperature centrifugation at 12,000 rcf for 10 minutes. Meanwhile, any remaining cytoskeletal elements in the wells were dissolved in the above buffer with addition of 0.5% SDS. Finally, supernatants were transferred to a new tube and pellets re-suspended in the SDS-containing buffer with the remaining insoluble tubulins. Equal volumes were loaded for immunoblotting as described above.

### Microtubule recovery assay

To visualize microtubule regrowth, microtubules were first depolymerized by treatment with 20 μM nocodazole for 4 hours and then allowed to regrow following washout for specified periods of time. To ensure accurate washout times all coverslips for each cell line were initially contained within the same 10 cm dish which was then washed four times with fresh media before returning to media that had been pre-equilibrated in the cell incubator to 5% CO_2_ for regrowth in normal culture conditions. At desired time points, coverslips were removed for processing. Cells were stained for F-actin and microtubules as previously described^66^. In brief, cells were Triton-extracted before fixing in glutaraldehyde, which was then neutralized with sodium borohydride before blocking in horse serum and incubating with rhodamine phalloidin and FITC-conjugated anti-α-tubulin (Sigma, clone DM1a). The α-tubulin isoform has been shown to be unchanged with Taxol-resistance^10^.

### EB3 microtubule tracking

To visualize live-cell microtubule dynamics, cells were transfected with mApple-EB3, a gift from Michael Davidson (Addgene plasmid #54892), using the TransIT-LT1 transfection reagent (Mirus Bio) per manufacturer's instructions. Cells were imaged with a Nikon Eclipse Ti inverted epifluorescent microscope, maintained at 37°C and 5% carbon dioxide throughout the experiment using an In Vivo Scientific environmental cell chamber and Bioscience Tools CO_2_ controller. A Photometrics QuantEM CCD camera (Princeton Instruments) was used to minimize exposure time while imaging with a Nikon CFI Apochromat TIRF 100X oil-immersion lens. Videos were captured at 1 Hz for 2 minutes and quantified using the open source u-track software as described^27^,^67^.

### Traction force cytometry

Cell-exerted forces were quantified by traction force cytometry as previously described^68^,^69^ by culturing cells on an elastic 10 kPa collagen-coated polyacrylamide substrate embedded with 200 nm fluorescent nanoparticles as fiduciary tracers. After capturing an initial image of the cells and particles, cells were removed by trypsinization and a final particle image captured. The embedded beads were used to determine gel displacement, which could then be used to calculate forces^70^.

### Statistics

The data are reported as mean ± standard error of the mean (SEM) from three independent experiments unless otherwise noted. Statistical analysis was carried out using a student's t-test or analysis of variance (ANOVA) followed by post-hoc analysis with Student–Newman–Keuls test, considering p < 0.05 to be significant (***p < 0.001,**p < 0.01,*p < 0.05). Pearson correlation coefficients (ρ), ranging from -1 for perfectly inversely correlated to +1 for perfectly positively correlated, were determined in MATLAB.

## Author Contributions

D.M., N.K., M.Q., K.P., N.R. and C.B. conducted experiments. D.M. designed experiments and analyzed data. D.M. and M.D. wrote the paper.

## Supplementary Material

Supplementary Information Supplementary Information

Supplementary Information Movie S1

Supplementary Information Movie S2
