# Supplementary material for: Alterations in Ovarian Cancer Cell Adhesion Drive Taxol Resistance by Increasing Microtubule Dynamics in a FAK-dependent Manner
Source: Sci Rep. 2015 Apr 17;5:9529. doi: 10.1038/srep09529 (PMC4400875; doi:10.1038/srep09529)
Supplement: Supplementary Information [file srep09529-s1.pdf]

# Alterations in Ovarian Cancer Cell Adhesion Drive Taxol Resistance by Increasing Microtubule Dynamics in a FAK-dependent Manner

Daniel J. McGrail,[1] Niti N. Khambhati,[2] Mark X. Qi,[1] Krishan S. Patel,[1] Nithin Ravikumar,[1] Chandler P. Brandenburg,[2] and Michelle R. Dawson[1,3*]

[1]School of Chemical & Biomolecular Engineering, Georgia Institute of Technology, Atlanta, GA
[2]School of Chemistry & Biochemistry, Georgia Institute of Technology, Atlanta, GA
[3]The Petit Institute for Bioengineering and Bioscience, Georgia Institute of Technology, Atlanta, GA

***Corresponding Author:**
Michelle Dawson, Ph.D.
School of Chemical & Biomolecular Engineering
Georgia Institute of Technology
311 Ferst Dr., N.W.
Atlanta, GA 30332-0100
Office: (404) 894-5192
Email: mdawson@gatech.edu
Website: http://dawson.chbe.gatech.edu/

**Running Title:** Taxol resistance alters ovarian cancer adhesion

**Figure S1.** OVCAR3 cells display decreased polymerized microtubules.

**Figure S2.** Adhesion differences are conserved across multiple ECMs.

**Figure S3.** Correlation of Taxol sensitivity with various parameters.

**Figure S4.** Alterations in focal adhesions in Taxol-resistant cells.

**Figure S5.** Increased Traction Forces in Taxol-resistant cells.

**Figure S6.** Full-length blots from manuscript.

**Video 1.** Video of mCherry-EB3 in parental SKOV3 cells

**Video 2.** Video of mCherry-EB3 in Taxol-resistant SKOV3 cells.

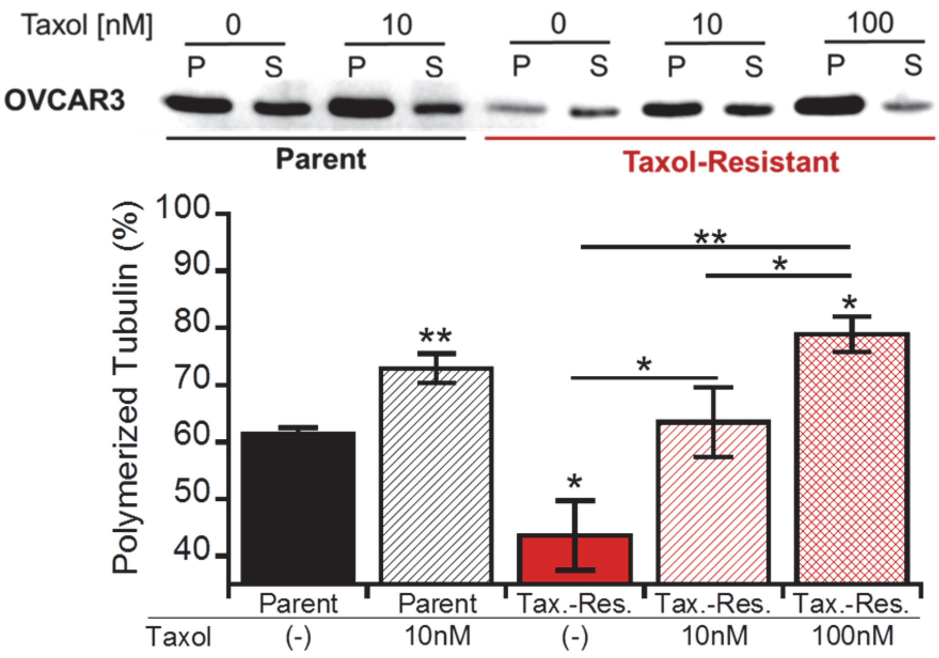


**Figure S1.**

**OVCAR3 cells display decreased polymerized microtubules.** Cells were lysed following 4 hour pretreatment with Taxol and separated into polymerized (P) and soluble (S) tubulin fractions for Western blot analysis. Percent polymerized tubulin was quantified as polymerized tubulin divided by the sum of polymerized and soluble tubulin. Values reported as mean ± SEM; significance indicated relative to control parent population unless otherwise noted, $*P<0.05$, $**P<0.01$,$***P<0.001$.

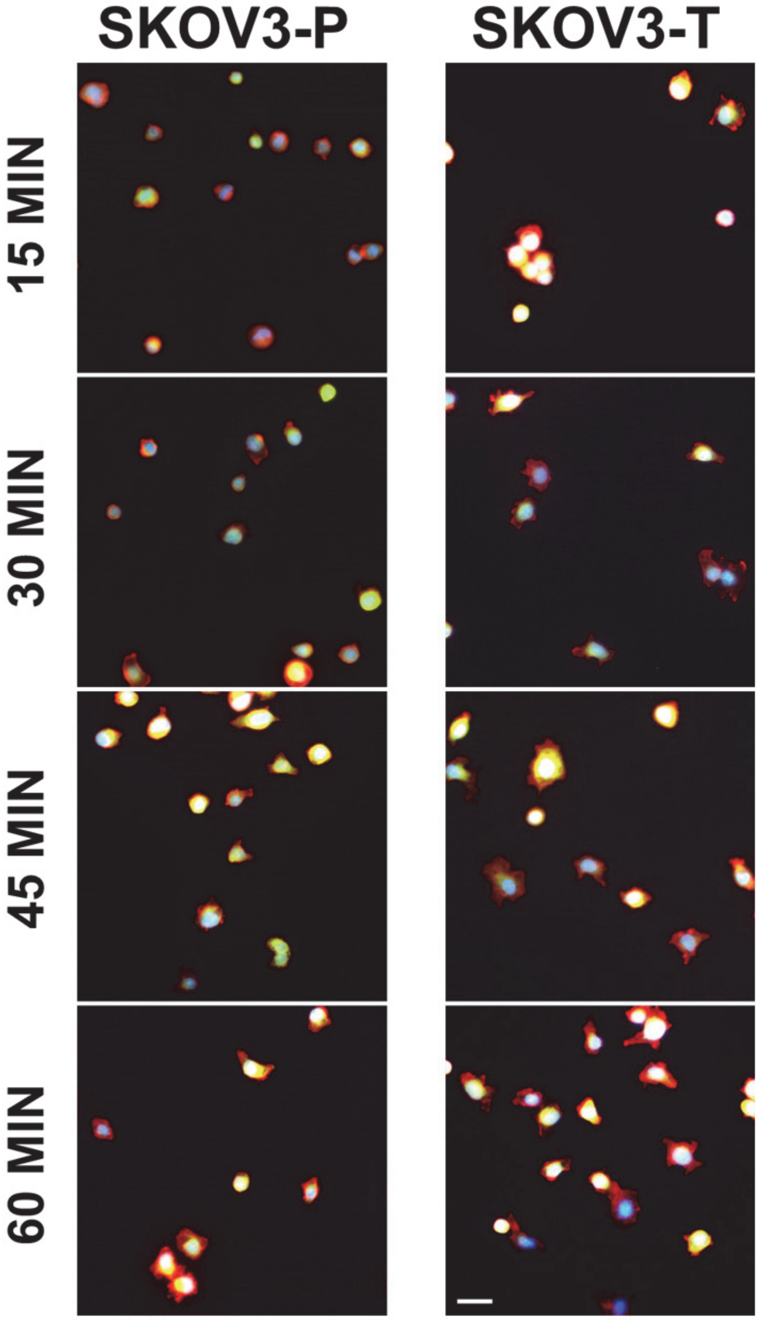


**Figure S2.**

**Cells spreading on collagen I.** Calcein-labeled cells were allowed to adhere to collagen I for specified length of time before directly fixing and staining for F-actin with Rhodamine Phalloidin (red) and nuclei with DAPI (blue). Scale bar = 20 μm.

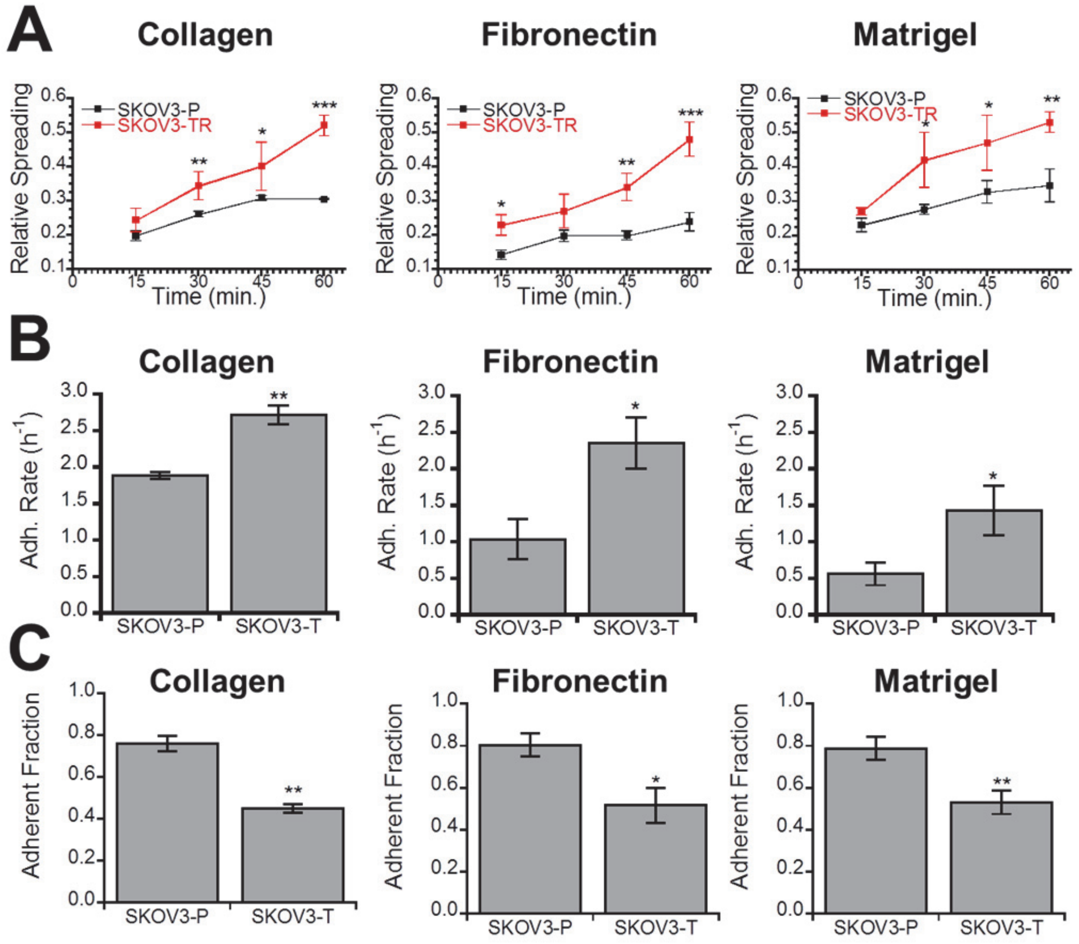


**Figure S3.**

**Adhesion differences are conserved across multiple ECMs.** Plates were coated either with 10 µg/mL Type I Collagen, 10 µg/mL fibronectin, or 100 µg/mL Matrigel and blocked with 1% heat-denatured BSA. **(A)** Cell spreading was analyzed by plating Calcein-labeled cells for specify period of time before fixing and staining F-actin with Rhodamine Phalloidin and nuclei with DAPI to quantify cell area (see Fig. S2). **(B)** Adhesion rate to various ECMs shows some baseline variation based on ECM, but increased adhesion rate in Taxol-resistant cells was conserved on all tested molecules. **(C)** Adhesion strength was also tested on all three ECMs with Taxol-resistant cells being more weakly adherent in all cases and no significant dependence on ECM. Values reported as mean ± SEM of three independent experiments, *$P<0.05$, **$P<0.01$,***$P<0.001$.

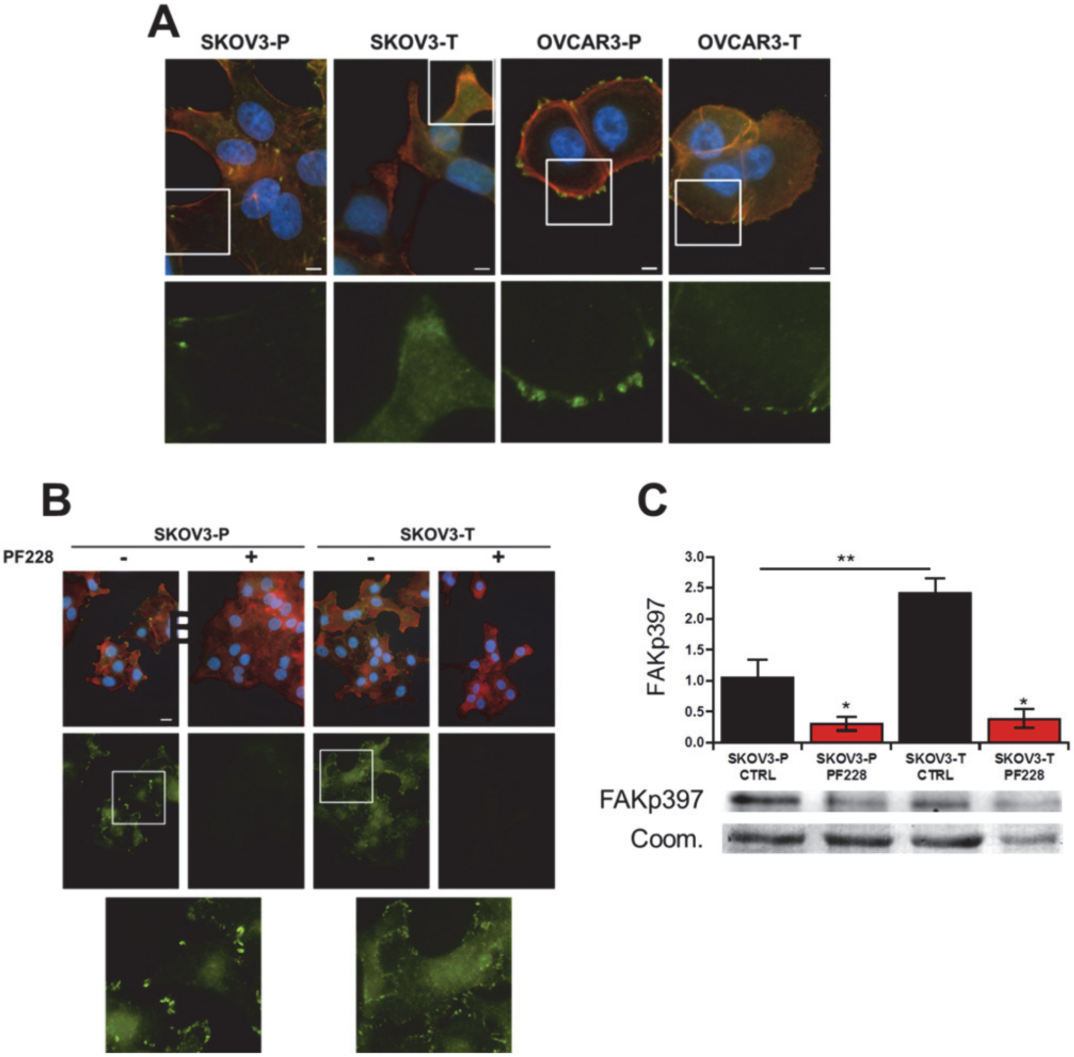


**Figure S4.**

**Alterations in focal adhesions in Taxol-resistant cells. (A)** Cells were stained for vinculin (green), actin (red) and nuclei (blue). While OVCAR displayed several large vinculin positive adhesions in the parental population and small vinculin positive adhesions in the Taxol-resistant population, only parental SKOV3 cells showed any formation of vinculin adhesions with diffuse staining in the Taxol-resistant population. **(B)** Cells were stained for FAKp397 (green), actin (red) and nuclei (blue). Consistent with other stains, this revealed large focal adhesions in the parent population with smaller focal adhesions in the Taxol-resistant cells coinciding with an increase in diffuse cytoplasmic signal. Staining was largely negligible after four hour incubation with 10 μM PF228. Scale bars = 10 μm. **(C)** Increased FAKp397 in Taxol-resistant cells demonstrated by Western blot, with inhibition to equivalent levels following four hour incubation with 10 μM PF228. Values reported as mean ± SEM of three independent experiments. Significance indicated relative to untreated control unless otherwise noted; *P<0.05, *P<0.01.

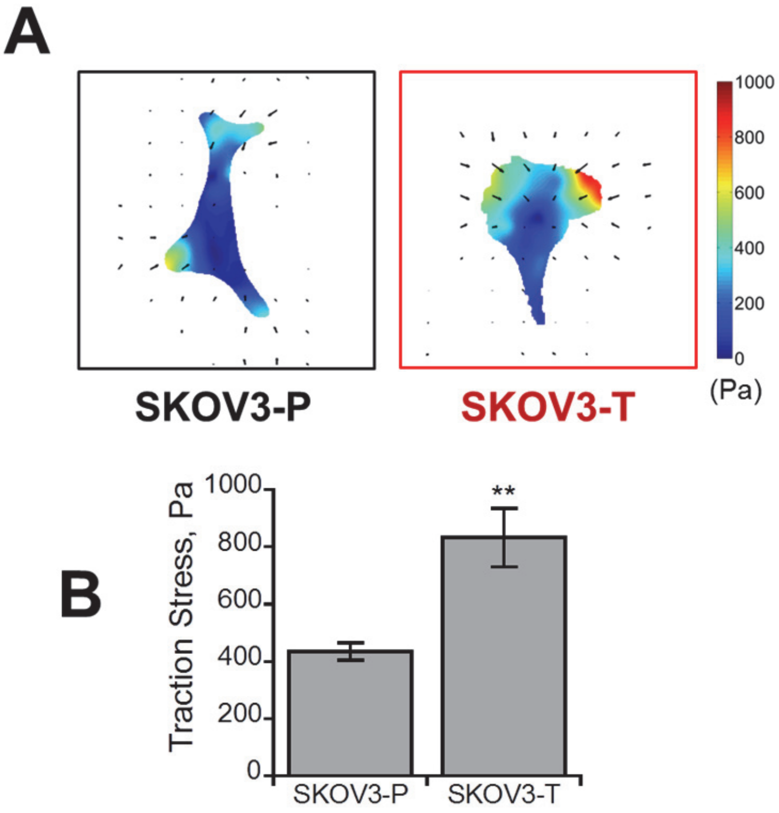


**Figure S5.**

**Increased Traction Forces in Taxol-resistant cells. (A)** Heat maps of traction stresses in Pascals show larger forces in Taxol-resistant cells. **(B)** Quantification of peak stress shows a two-fold increase in traction forces as quantified by average peak traction stress (N=3). Values given as mean ± SEM; *$P$<0.05, **$P$<0.01,***$P$<0.001.

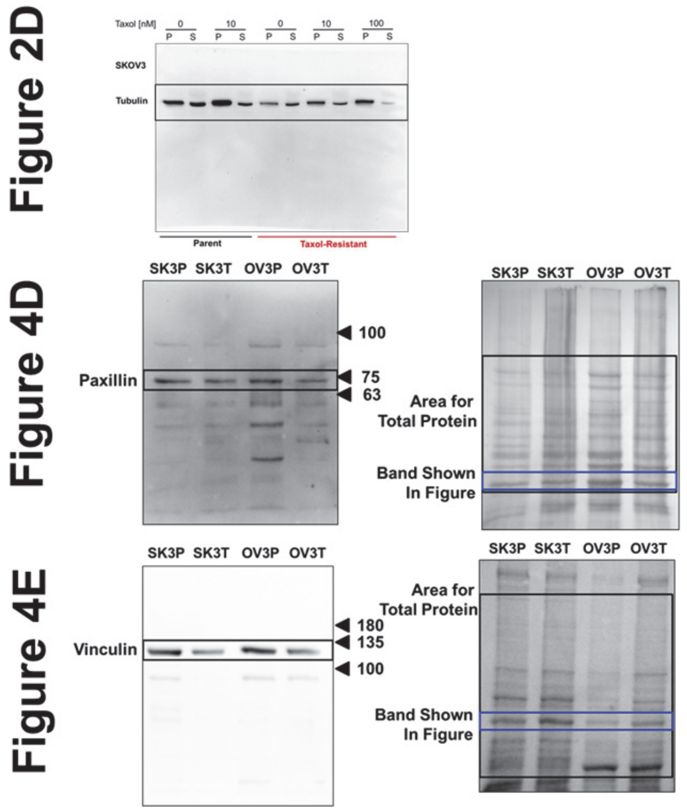


**Figure S6.**

Images of full-length blots from manuscript. Areas used for quantification are outlined in black.
